# Genetic Diversity of Prolamin Loci Related to Grain Quality in Durum Wheat (*Triticum durum* Desf.) in Kazakhstan

**DOI:** 10.3390/life16010157

**Published:** 2026-01-17

**Authors:** Maral Utebayev, Svetlana Dashkevich, Oksana Kradetskaya, Irina Chilimova, Ruslan Zhylkybaev, Tatyana Zhigula, Tatyana Shelayeva, Gulmira Khassanova, Kulpash Bulatova, Vladimir Tsygankov, Marat Amangeldin, Yuri Shavrukov

**Affiliations:** 1 A.I. Barayev Research and Production Centre of Grain Farming, Shortandy 021601, Kazakhstan; vetka-da@mail.ru (S.D.); oksana_cwr@mail.ru (O.K.); coronela@mail.ru (I.C.); gaziz-amonik@mail.ru (R.Z.); tanya.zhigula@mail.ru (T.Z.); tatyana.shelaewa@yandex.kz (T.S.); 2 Faculty of Agronomy, S. Seifullin Kazakh AgroTechnical Research University, Astana 010000, Kazakhstan; khasanova-gulmira@mail.ru; 3 Kazakh Research Institute of Agriculture and Plant Growing, Almalybak 040909, Kazakhstan; bulatova_k@rambler.ru; 4 Kazakh Research Institute of Horse Breeding and Fodder Production, Aktobe 030014, Kazakhstan; zigan60@mail.ru; 5 Faculty of Biology and Biotechnology, Al-Farabi Kazakh National University, Almaty 050040, Kazakhstan; marat.amangeldin@gmail.com; 6 College of Science and Engineering, Biological Sciences, Flinders University, Adelaide, SA 5042, Australia

**Keywords:** allele variants, durum wheat, electrophoresis, gliadin, HMW and LMW glutenin subunits, quality of grain

## Abstract

The technological properties of durum wheat grain are determined by prolamins (gliadins and glutenins). Information on the allelic composition of key loci remains incomplete despite existing global studies examining prolamin variability. This highlighted the need to study these traits in durum wheat in Kazakhstan. The effects of specific gliadin components with high- and low-molecular-weight glutenin fractions on gluten quality are also not fully clarified. This study aimed to characterise allelic diversity at prolamin-coding loci and evaluate associated grain quality traits. Using native and denaturing SDS-electrophoresis, 181 tetraploid wheat accessions from Kazakhstan, an International germplasm collection, and 26 breeding lines were analysed for allelic variation and associations with protein content, gluten content, gluten index, and SDS-sedimentation. The *γ45* gliadin component and *Glu-A3a* allele were positively associated with SDS-sedimentation and gluten index, while *Glu-B3b* had a negative effect. Distinct prolamin profiles were observed among accessions from different ecological and geographical locations. These results support the selection of superior durum wheat genotypes and enable the identification of favourable allele combinations at the *Gli-1*, *Gli-2*, *Glu-1*, and *Glu-3* loci in cultivars from Kazakhstan. Comparison with global tetraploid wheat germplasm collections demonstrates unique genetic diversity in genotypes, providing a valuable basis for breeding programs aimed at improving grain and gluten quality in durum wheat in Kazakhstan and Central Asian countries.

## 1. Introduction

Durum wheat (*Triticum durum* Desf.) is one of the most important cereal crops worldwide, accounting for approximately 8% of the global wheat-growing area [1]. Its grain is widely used for pasta, semolina, and various traditional foods, which creates high and stable demand for grain with specific technological properties [2].

Consequently, grain quality remains a key target in durum wheat breeding programs. Grain quality in durum wheat is largely determined by the gluten protein complex, composed of gliadins and glutenins. Gliadins are encoded primarily by the *Gli-1* and *Gli-2* loci, resulting in extensive allelic variation. Glutenins are classified into high- and low-molecular-weight subunits (HMW-GS and LMW-GS), encoded mainly by the *Glu-1* and *Glu-3* loci, respectively. Both groups contribute to gluten strength and viscoelastic properties, although their relative effects differ depending on allelic composition and genetic background [3]. Strong associations between specific gliadin components and gluten quality have been demonstrated, particularly at the *Gli-B1* and *Glu-B3* loci [4,5,6,7,8]. Extensive catalogues of HMW-GS and LMW-GS allelic variants now enable large-scale assessment of prolamin diversity in international durum wheat collections [9,10,11,12]. Previous studies have reported substantial geographic variation in the allelic composition of gliadin- and glutenin-encoding loci in durum wheat; however, available data remain fragmented across countries [13,14,15,16,17,18,19] and are largely absent for Kazakhstan.

Therefore, there is a clear need to conduct research providing a comprehensive account of the diversity of gliadin- and glutenin-encoding loci in durum wheat in Kazakhstan. The aim of the present study was to comprehensively characterise the allelic diversity of gliadin- and glutenin-encoding loci (HMW-GS and LMW-GS) in a representative collection of durum wheat germplasm from Kazakhstan and to identify favourable prolamin alleles and allele combinations that can be exploited in durum wheat breeding programs.

## 2. Materials and Methods

### 2.1. Plant Material

A total of 181 durum wheat accessions and 26 breeding lines from Kazakhstan and global breeding programs were analysed. Seeds were provided by the Genetic Resources Laboratory, A.I. Barayev Research and Production Centre of Grain Farming (BRPCGF). Wild species included *Triticum turanicum*, *T. persicum*, *T. sovieticum*, *T. dicoccum*, and *T. aethiopicum* Jakubz. In addition, wild accessions from field expeditions, such as white beardless spelt (France), Korasim in the Upper Jordan Valley 1972 (Israel), and Rosh-Pinnar in East Galilee (Israel), were provided by the Australian Grains Genebank, Horsham (Australia). The full list of durum wheat accessions used in this study is present in Supplementary Materials Tables S1 and S2. A subset of 26 durum wheat breeding lines, selected to study associations between prolamin alleles and grain quality, was sown at the research field of BRPCGF, Akmola region, Kazakhstan (51°40′25.3″ N, 71°0′46.8″ E). Each line was grown in a seven-row plot, 25 m in length, 1 m in width, with a 25 m^2^ area. Seeds were sown to a depth of 5–7 cm, with 10–12 cm spacing between rows, and about 280 plants per m^2^. Each genotype had two replicated plots with a fully randomised design. The soil was chestnut chernozem without irrigation.

### 2.2. Weather Conditions of the Growing Seasons

In 2022–2024 experiments, wheat plants were grown in Northern Kazakhstan in contrasting agro-climatic conditions with variability in temperature and moisture. According to regional agro-meteorological observations, the long-term average precipitation during the wheat growing season (May–August) in Northern Kazakhstan was approximately 180–200 mm, and air temperature 16.5–17.5 °C. In 2022, precipitation in the same period was below the long-term average with a hydrothermal coefficient (HTC) of about 0.4–0.5, indicating a moderate drought. In 2023, the growing season was characterised as a severe drought with HTC values dropping to 0.1–0.2. Precipitation was below 120 mm, and air temperatures exceeded the long-term average by 1.5–2.0 °C. In contrast, 2024 had a much higher moisture availability, with precipitation exceeding 230 mm and HTC values reaching 1.3–1.5, corresponding to favourable conditions for wheat growth and grain filling.

### 2.3. Biochemical Analysis

Protein content (PC) was determined by the Kjeldahl method using an automatic distillation unit UDK 142 (Velp Scientifica, Usmate‑Velate, Italy). Gluten content (GC) and gluten index (GI) were measured with a Glutomatic 2200 system (Perten Instruments, Hägersten, Sweden), following the manufacturer’s instructions. The results of the SDSS test were obtained through sedimentation of ground grain in lactic acid [20], with some modifications [21].

### 2.4. Prolamin Extraction

Gliadins were extracted according to the following protocol: wheat kernels were milled, and gliadins were extracted for 12 h at room temperature using 70% ethanol in microtube. Subsequently, a staining solution based on methylene green was added, prepared as follows: 60.0 g of sucrose and 0.01 g of methylene green were dissolved in 100 mL of 5.1 mM aluminum lactate buffer at pH 3.1.

Glutenin extraction was performed based on the proposed method [22], with several modifications. The crushed seed was placed in a 2.0 mL Eppendorf tube, 350 µL of 70% ethanol was added, and the samples were incubated at 65 °C for 45 min. The tubes were then centrifuged at 12,000× *g* for 5 min, the supernatant was discarded, and the pellet was washed with 400 µL of 70% ethanol, followed by centrifugation under the same conditions. The pellet was dried to remove residual ethanol, and 300 µL of extraction buffer was added. The extraction buffer composition was: 2.0 g SDS–Na, 10.0 mL glycerol, 80 mL Tris-HCl buffer (pH 8.8), 1.0 mg bromophenol blue, brought to a final volume of 100 mL with distilled water. Prior to extraction, β-mercaptoethanol was added to the buffer at a ratio of 500 µL per 10 mL of extraction buffer.

### 2.5. Gliadin and Glutenin Allele Classification

Gliadin electrophoresis was carried out according to the procedure described earlier [23]. The separation of HMW-GS and LMWGS was performed in 12% polyacrylamide gels using denaturing SDS-PAGE [24], with minor modifications [25]. The durum wheat cultivars Langdon and Senatore Capelli (*Triticum durum* Desf.), as well as bread wheat reference cv. Chinese Spring (*Triticum aestivum* L.), were used as standards for the identification of durum wheat genotypes (cultivars and breeding lines) based on catalogues for gliadin and glutenin alleles [10,26,27]. The nomenclature of gliadin and glutenin loci used for durum wheat was based on the Gene Catalogue for bread wheat gliadins: *Gli-A1*, *Gli-B1*, *Gli-A2*, and *Gli-B2*; and glutenins: *Glu-A1*, *Glu-B1*, *Glu-A3*, *Glu-B3*, and *Glu-B2* [28]. Alleles at each locus were designated by Latin letters as a ‘genetic formula’ in the same order of genes. For instance, the genetic formula of gliadins in cv. Langdon was: *Gli-A1c*, *Gli-B1a*, *Gli-A2a*, and *Gli-B2a*, with the following final abbreviation: *c*, *a*, *a*, *a*.

### 2.6. Statistical Treatment

In this study, various methods were used to assess population diversity and inter-population differences based on the allelic composition of prolamins. The use of multiple parameters to describe diversity is justified, as each coefficient characterizes a specific aspect of genetic variation and complements the overall picture. Genetic diversity within populations was evaluated using the mean number of alleles per locus (*μ*), the proportion of rare alleles (*h*) [29], and Nei’s gene diversity index (*H*) [30]. Allele frequencies for each locus were determined based on the observed allele counts in individual samples. Genetic similarity between populations of different geographic origins was assessed using the similarity index (*r*). The statistical significance of differences between populations was evaluated using the identity criterion (*I*), comparing its value with the *χ^2^* distribution at a significance level of *p* < 0.01 [29].

Associations between alleles at different loci were analyzed using Pearson’s coefficient of association with Yates’ correction and Yule’s coefficient [31]. The significance of allelic associations was evaluated using the *χ^2^* test with one degree of freedom:

(1) $$r_{A}=\frac{\left(\left|ad-bc\right|\right)-0.5N}{\sqrt{\left(a+b)(a+c)(b+d)(c+d\right)}}$$

and

(2) $$\chi^{2}=N\cdot r_{A}^{2}$$

If $\chi^{2}>\chi_{St}^{2}$ for k = 1, then the relationship is considered significant. Yule’s association coefficient [31]:

(3) $$r_{Q}\;=\;\frac{ad-bc}{ad+bc}$$

The statistical error of the Yule coefficient is calculated using the formula:

(4) $$S_{r_{Q}}=\frac{1-r_{Q}^{2}}{2}\sqrt{\frac{1}{a}+\frac{1}{b}+\frac{1}{c}+\frac{1}{d}}$$

Differences in mean values between groups were assessed using Welch’s *t*-test, which does not assume equal variances [32]. Results were considered statistically significant at *p* < 0.05:

(5) $$t\;=\;\frac{{\bar{x}}_{1}-{\bar{x}}_{2}}{s_{\left({\bar{x}}_{1}-{\bar{x}}_{2}\right)}}=\frac{d}{s_{d}}$$

where

(6) $$s_{d}=\sqrt{\frac{\sum (x_{1}-{\bar{x}}_{1}{)}^{2}+\sum (x_{2}-{\bar{x}}_{2}{)}^{2}}{\left(n_{1}-1\right)+\left(n_{2}-1\right)}(\frac{n_{1}+n_{2}}{n_{1}n_{2}})}$$

All statistical analyses were conducted using Microsoft Excel 2021. For multivariate analysis, allele frequency data were standardized, and clustering was performed using Ward’s method with Euclidean distances in Statistica 6.0 (StatSoft, Inc., Tulsa, OK, USA).

## 3. Results

Based on electrophoresis of gliadins and glutenins, alleles of *Gli-1*, *Gli-2*, *Glu-1*, and *Glu-3* loci were identified and presented as genetic formulas in Supplementary Material Table S1. The characteristics of the allelic composition of gliadins and glutenins in tetraploid wheat from Kazakhstan (*N* = 86) and in the world collection (*N* = 95) will be discussed in the following sub-sections separately.

### 3.1. Tetraploid Wheat Collection from Kazakhstan

#### 3.1.1. *Gli-1* and *Gli-2* Loci

Gliadin-coding loci (*Gli-1* and *Gli-2*) exhibited multiple allelism, reflecting genetic polymorphism within the collection. In 86 tetraploid wheat accessions from Kazakhstan, the highest polymorphism was observed at the *Gli-B2* locus (11.6%), followed by *Gli-B1* (8.1%), *Gli-A2* (7.0%), and *Gli-A1* (5.8%). Overall, 17.4% of accessions carried rare gliadin alleles (15 out of 86).

**Figure 1:**
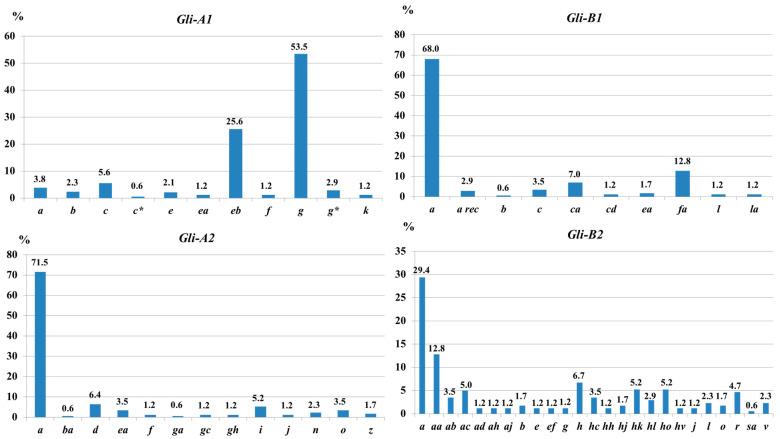
Allelic frequency (*%*) in studied loci of gliadin, *Gli-1* (*Gli-A1* and *Gli-B1*), and *Gli-2* (*Gli-A2* and *Gli-B2*), in durum wheat accessions from Kazakhstan (*N* = 86). Two allelic variants of gliadin, *c* and *g*, are designated using additional asterisks as follows: c* and g*.

Accessions with identical sets of gliadin alleles were often distinguished by differences in LMW-GS composition, confirming their distinct genotypes (e.g., Hordeiforme 207-92: *Glu-A3a*, *Glu-B3b*, *Glu-B2b*; Nauryz-3: *Glu-A3h*, *Glu-B3c*, *Glu-B2b*; see Supplementary Material Table S1). The number of alleles per locus ranged from 10 (*Gli-B1*) to 25 (*Gli-B2*), whereas for loci *Gli-A1* and *Gli-A2*, the number of alleles was 11 and 13, respectively. In particular, *Gli-A1g* (53.5%) and *Gli-A1eb* (25.6%) were most frequent at *Gli-A1*, whereas *Gli-B1a*, *Gli-A2a*, and *Gli-B2a* dominated their respective loci (Figure 1). Detailed allelic formulas are presented in Supplementary Material Table S1.

#### 3.1.2. *Glu-1* Loci

**Figure 2:**
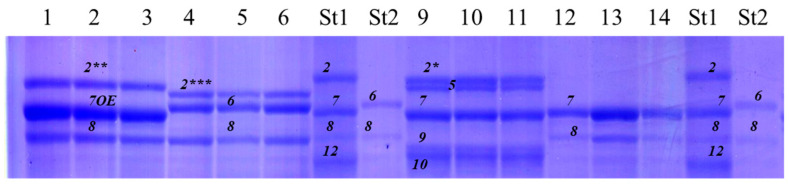
Separation of high-molecular-weight glutenin subunits of durum wheat in 12% polyacrylamide gel using denaturing SDS-PAGE. Genotypes: 1–3, Kostanayskaya-30; 4–6, Kargala-16; St1, Chinese Spring (*Triticum aestivum* L.); St2, Langdon (*Triticum durum* Desf.); 9–11, Neepawa (*Triticum aestivum* L.); 12–14, Kargala-24. Two allelic variants of the high-molecular-weight glutenin subunit 1Ax2* are designated using additional asterisks as follows: 1Ax2** and 1Ax2***.

**Figure 3:**
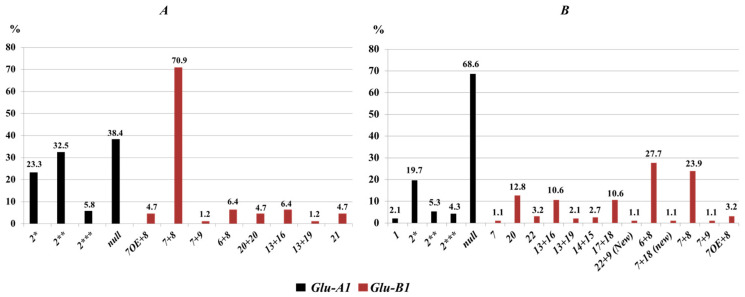
Frequency (%) of high-molecular-weight glutenin subunits of *Glu-1* loci in durum wheat. (**A**) Local durum wheat germplasm collection from Kazakhstan (*N* = 86); (**B**) International tetraploid wheat germplasm collection (*N* = 94). Two allelic variants of the high-molecular-weight glutenin subunit 1Ax2* are designated using additional asterisks as follows: 1Ax2** and 1Ax2***.

Allelic diversity at HMW glutenin (*Glu-1*) loci in durum wheat from Kazakhstan was lower than at gliadin loci, consistent with the absence of D genome-encoded proteins in the tetraploid genome. Identified HMW-GS subunits and corresponding alleles are listed in Supplementary Material Table S1. Two additional subunits, 1Ax2** and 1Ax2*** (Figure 2), exhibited higher electrophoretic mobility than the classical 1Ax2* subunit (cv. Neepawa, bread wheat) and likely represent allelic variants of 1Ax2*. Predominant subunits were determined based on their frequency of occurrence (Figure 3).

The *Glu-B1* locus comprised eight alleles. *Glu-B1b*, encoding the 1Bx7 + 1By8 subunit pair, was most frequent (70.9%). The *Glu-B1al* allele, present in 4.7% of accessions, encodes 1Bx7^OE^ + 1By8 and is of particular breeding interest (Figure 2). Overall, 33.7% of accessions (29/86) carried *Glu-1* variants. *Glu-A1* was more diverse than *Glu-B1* (27.9%, 24/86), primarily represented by 1Ax2* (b) and the null allele (c). Polymorphism at the *Glu-B1* locus was 11.6% (10/86), largely due to the combination of *Glu-B1b* with other alleles.

#### 3.1.3. *Glu-3* Loci

**Figure 4:**
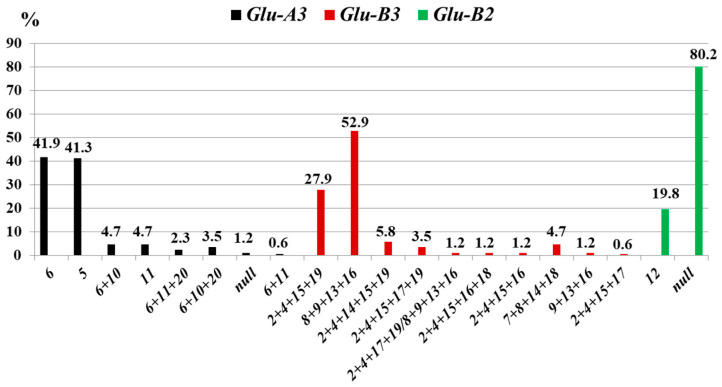
Frequency (*%*) of low-molecular-weight glutenin subunits at the *Glu-3* loci in durum wheat from Kazakhstan (*N* = 86).

At the *Glu-A3* locus, eight alleles were identified in the studied collection. The most frequent alleles were *a* (41.9%) and *b* (41.3%), which are associated with the synthesis of LMW glutenin subunits 6 and 5, respectively (Figure 4).

**Figure 5:**
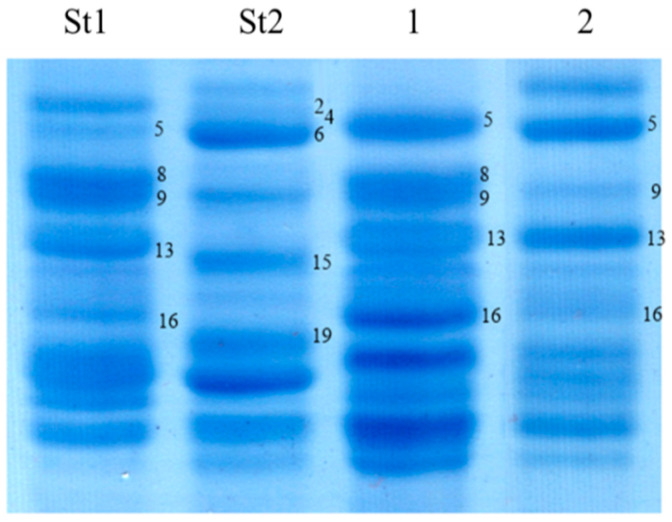
Low-molecular-weight glutenin subunits in durum wheat from Kazakhstan. St1, standard cv. Langdon; St2, standard cv. Senatore Capelli; 1, Breeding line 654.1-2-3-4; 2, cv. Kargala-28.

The *Glu-B3* locus was represented by ten allelic variants. The predominant allele, Glu-B3b, controlled the synthesis of LMW glutenin subunits 8 + 9 + 13 + 16, observed in 52.9% of accessions. The *Glu-B3a* allele, encoding subunits 2 + 4 + 15 + 19, was found in 27.9% of accessions. Comparison of LMW glutenin subunit combinations with previously published allelic diagrams [10] indicated partial correspondence. For instance, the *Glu-B3b* allele is expected to produce four subunits (8 + 9 + 13 + 16). In the present study, three of these four subunits were detected in cv. Kargala-28, consistent with the allele identification based on electrophoretic mobility (Figure 5, lane 2).

Similarly, in cv. Kargala-69, an LMW glutenin combination resembling the pattern of *Glu-B3d*, was observed; however, subunit 15 was absent. These observations highlight intra-allelic variability and partial expression of expected subunits, emphasizing that electrophoretic patterns may not always fully correspond to previously reported allelic formulas.

### 3.2. International Tetraploid Wheat Germplasm Collection

#### 3.2.1. *Gli-1* and *Gli-2* Loci

Based on gliadin electrophoresis and the derived genetic formulas (Supplementary Material Table S1), the level of genetic polymorphism was as follows: *Gli-B1*, *Gli-A2*, and *Gli-B2* loci showed polymorphism in 4 out of 95 accessions (4.2%), while *Gli-A1* was polymorphic in 3.2% of samples. Overall, 6 out of 95 accessions (6.3%) carried rare gliadin alleles. The number of identified alleles per locus was: *Gli-A1*–15, *Gli-B1*–19, *Gli-A2*–22, and *Gli-B2*–36 alleles (Supplementary Material Table S3). Specifically, two new blocks were identified for *Gli-B1* and *Gli-A2*, three for *Gli-A1*, and up to seven for *Gli-B2*. This variation may be attributed to the inclusion of wild tetraploid wheat accessions in the study. Within each locus, two or three alleles with the highest frequency predominated. The most frequent alleles were: *Gli-A1c* (29.8%), *Gli-A1b* (27.7%), *Gli-B1a* (27.7%), *Gli-A2a* (36.2%), *Gli-A2o* (25.0%), *Gli-B2a* (15.4%), and *Gli-B2hk* (13.8%) (Supplementary Material Table S3). Similar to the Kazakhstan collection, some genotypes shared identical gliadin compositions. For example, accessions Ld-12 and Ld-134 exhibited the combination: *Gli-A1g*, *Gli-B1a*, *Gli-A2a*, *Gli-B2a*. In these accessions, HMW glutenin subunits 1Bx7 and 1By8 were detected based on SDS-PAGE electrophoretic mobility. In Ld-12, the 1Bx7 band exhibited higher intensity, consistent with its putative identification as the allelic variant 1Bx7^OE^. Analysis of LMW glutenin subunits revealed allelic differences between these genotypes. Accession Ld-134 carried *Glu-A3b*, *Glu-B3b*, and *Glu-B2b*, whereas Ld-12 had *Glu-A3b*, *Glu-B3i*, and *Glu-B2b*. These results indicate allelic variation at the *Glu-B3* locus between Ld-12 and Ld-134, despite identical gliadin profiles.

#### 3.2.2. *Glu-1* Loci

Similarly to the Kazakhstan durum germplasm collection, HMW glutenin spectra revealed the allelic variant of the 1Ax2* subunit (Figure 3). The total frequency of these subunits was 29.3%, whereas the functional null allele (*Glu-A1c*) occurred in 68.6% of accessions, nearly twice the frequency observed in the Kazakhstan collection. The 1Ax1 subunit was detected in only two accessions: Grecale (Italy) and Tibetka (Kyrgyzstan).

**Figure 6:**
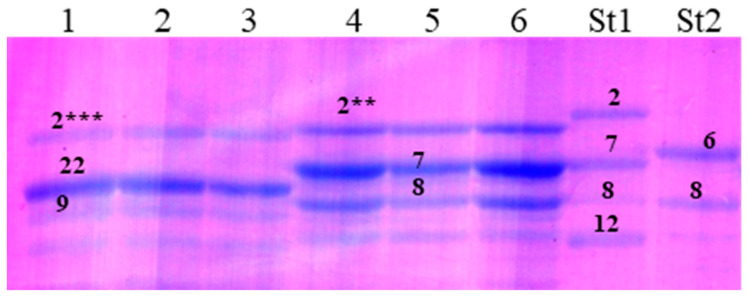
High-molecular-weight glutenin subunits in durum wheat from the International germplasm collection. Lanes 1–3, IG-85503 (Pakistan); 4–6, PI-388132 (Pakistan); St1, bread wheat cv. Chinese Spring (*Triticum aestivum* L.); St2, durum wheat cv. Langdon (*Triticum durum* Desf.). Two allelic variants of the high-molecular-weight glutenin subunit 1Ax2* are designated using additional asterisks as follows: 1Ax2** and 1Ax2***.

At the *Glu-B1* locus, 13 HMW glutenin subunit combinations were identified. The most frequent were 1Bx6 + 1By8 (27.4%) and 1Bx7 + 1By8 (23.7%). Several combinations differed from the classical and supplemental HMW-GS nomenclature [27,33]. For example, in accession IG-85503 (Pakistan), a subunit pair identified by SDS-PAGE as 22x + 9 (Figure 6) was substantially different from classical subunits 9 and 22, representing a putative assignment based on electrophoretic mobility. Overall, polymorphism at glutenin-coding loci was low. At *Glu-A1*, only 2 of 95 accessions (2.1%) were polymorphic, and at *Glu-B1*, 1 of 95 accessions (1.1%) showed variation.

#### 3.2.3. *Glu-3* Loci

**Figure 7:**
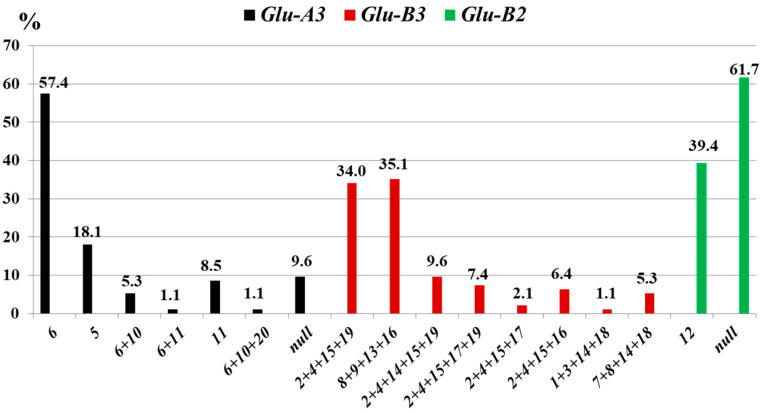
Frequency of low-molecular-weight glutenin subunits (*%*) at the *Glu-3* loci in tetraploid wheat accessions from the International germplasm collection (*N* = 94).

Using denaturing SDS-PAGE, seven variants of LMW glutenin subunits encoded by the *Glu-A3* locus and eight subunit combinations encoded by the *Glu-B3* locus were identified (Figure 7). The highest frequencies were observed for subunit 6 of the *Glu-A3a* allele (56.8%), as well as for the subunit combination 2 + 4 + 15 + 19 of the *Glu-B3a* allele (33.7%) and the combination 8 + 9 + 13 + 16 of the *Glu-B3b* allele (34.7%).

The majority of accessions from the international germplasm collection exhibited a null variant at the *Glu-B2* locus.

### 3.3. Statistical Analysis

Based on statistical calculations, the proportion of rare alleles (*h*), intra-population (*μ*), and genetic diversity (*H*) of tetraploid wheats from both the International and Kazakhstan germplasm collections are presented in Table 1. The intra-population diversity indicator (*μ*) characterises the population based on the number of rare alleles and their frequency. For example, quite close values of 3.66 ± 0.10 and 3.44 ± 0.05 were found for the *Glu-A1* locus in both germplasm collections. In the International germplasm collection, the null allele *Glu-A1c* predominated, with a frequency of 68.6%, whereas in the Kazakhstan collection, the frequency distribution of *Glu-A1* alleles was more balanced (Figure 3). Despite the higher number of identified HMW glutenin variants in the International collection, the within-population diversity index (*μ*) was lower, reflecting the dominance of the null allele.

The proportion of rare alleles (*h*) reflects the evenness of allele frequency distribution. For the *Glu-B1* locus, the h value was higher in wheat from Kazakhstan (0.44 ± 0.05) than in the international germplasm collection (0.26 ± 0.04), indicating a more uneven distribution of allele frequencies in the Kazakhstan population. In this collection, the subunit pair 1Bx7 + 1By8 predominated, whereas the remaining subunits occurred at much lower frequencies. In contrast, in the international durum wheat collection, subunit pairs 1Bx7 + 1By8 (23.9%) and 1Bx6 + 1By8 (27.7%) were observed at comparable frequencies, and other subunits also showed substantial occurrence, indicating a more even genotype distribution. The degree of genetic diversity (*H*) ranged from 0.32 at the *Glu-B2* locus to 0.93 at the *Gli-B2* locus. Overall, statistical analysis demonstrated that the international tetraploid wheat collection was characterized by higher allelic richness and genetic diversity (*H*) at most *Gli* loci, whereas the Kazakhstan collection exhibited a more uneven frequency distribution with a limited number of predominant alleles, particularly at the *Glu-1* loci. These differences reflect contrasting population structures shaped by breeding history and germplasm composition.

### 3.4. Biochemical and Technological Analyses and Allelic Composition of Prolamins in Durum Wheat Breeding Lines from Kazakhstan

Over three years, 26 durum wheat breeding lines showed a mean protein content (PC) of 15.4% and gluten content (GC) of 32.9%. Gluten index (GI) and SDS-sedimentation (SDSS) averaged 36 units and 35 mL, respectively (Supplementary Material Table S2), with 16 lines exhibiting normal gluten strength.

Gliadin electrophoresis revealed γ45 components in 19 of 26 genotypes. HMW glutenins at *Glu-A1* included 1Ax1 (46.2%) and Ax2* (36.5%), while *Glu-B1* was represented mainly by 1Bx6 + 1By8 (14.3%) and 1Bx7 + 1By8, including 1Bx7^OE^ (84.6%). LMW glutenins were dominated by subunit 6 (*Glu-A3a*, 67.3%) and subunit 5 *(Glu-A3b*, 23.1%) at *Glu-A3*, and by 8 + 9 + 13 + 16 (*Glu-B3b*, 48.1%) and 2 + 4 + 15 + 16 (*Glu-B3g*, 26.9%) *at Glu-B3*. The *Glu-B2* locus was largely represented by the null allele *Glu-B2b* (73%) (Supplementary Material Table S2). Correlation analysis indicated a positive relationship between GI and SDSS (*r* = 0.73 ± 0.14).

Associations between prolamin alleles and quality traits were evaluated using χ^2^ and Yule’s Q (Table 2). Preliminary optima of qualitative characteristics were compiled for the calculations: PC 13.5–15.5%; GC 28–35%; GI from 30 units or more; SDSS from 30 mm or more. *Glu-A3a* and γ45 showed positive effects on GI and SDSS, while *Glu-B3b* was associated with lower values.

Some loci, including *Glu-B2b* and 1Bx7 + 1By8, exhibited inconsistent trends across statistical tests, reflecting the polygenic nature of protein and gluten content. Given the limited sample size, associations not consistently supported by both *χ^2^* and Yule’s Q should be considered indicative and require validation in larger datasets.

## 4. Discussion

The present results provide a basis for the selection of durum wheat genotypes with desirable quality traits. Gliadin and glutenin profiles were analysed in cultivars and breeding lines produced by the main Kazakh Breeding Centres, including rare tetraploid accessions from germplasm collections. These genotypes are of breeding interest due to traits such as higher gluten and protein content, elevated carotenoids, and disease resistance. Their inclusion enabled assessment of genetic diversity at *Gli-1*, *Gli-2*, *Glu-1*, and *Glu-3* loci and evaluation of breeders’ preferences in allele combinations in Kazakhstan durum wheat relative to international tetraploid wheat.

For comparative analysis and statistical calculations, the tetraploid wheat accessions were grouped according to their eco-geographic origin. Six groups were formed: (1) Eurasian region: Kazakhstan, Russia, Kyrgyzstan, Armenia, China and Mongolia with 106 accessions; (2) Southwest and Southern Asia: Iraq, Iran, Syria, Pakistan, India, Afghanistan, Saudi Arabia, Israel and Palestine with 19 accessions; (3) North, East, and South Africa: Algeria, Ethiopia, Egypt, Morocco, Tunisia and South Africa with 14 accessions; (4) Australia with 6 accessions; (5) Southern, Western and Central Europe: Cyprus, Greece, Italy, Portugal, Spain, Switzerland, Austria and France with 23 accessions; (6) North and South America: USA, Mexico and Peru with 13 accessions. To obtain more detailed information on the genetic diversity of alleles encoding prolamin synthesis, previously published data were also used [8,9,11,13,16,18,34,35,36,37,38] (Supplementary Material Table S4). Loci *Gli-B5* and *Glu-B2* were not included in the comparative analysis, since the polymorphism of these genes was represented by only two allelic variants. In the current study, the characteristics of each gliadin- and glutenin-coding locus and the effects of their alleles are discussed separately.

### 4.1. Gli-1 and Gli-2 Loci

In the Kazakh tetraploid wheat collection, 59 of the 131 alleles were identified using the reference catalogue [26], confirming the comparability of the material with previously studied collections. The most prevalent alleles were *Gli-A1g*, *Gli-B1a*, *Gli-A2a*, and *Gli-B2a*, which are also typical of durum wheat cultivars from Russia, Ukraine, and China [14]. The *Gli-A1g* allele exhibited broad ecological plasticity and was detected in all geographic groups except the African one. The *Gli-A1c* allele, characteristic of Italian cultivars [39], was absent only from the Australian group, possibly reflecting the limited sample size. The *Gli-A1b* allele occurred in all six regions, reaching its highest frequencies in the African and European groups, and has previously been described as characteristic of Spanish durum wheat cultivars [39]. Alleles of the *Gli-1* locus associated with gluten content and quality, particularly *Gli-A1a* and *Gli-A1c* [40], were detected at low frequencies in the Kazakh wheat collection. Several alleles (*Gli-A1ab*, *Gli-A1f*, and *Gli-A1k*) were specific to the Eurasian group and, despite their low frequencies, contributed to the maintenance of genetic diversity.

The *Gli-B1* locus is of particular interest due to its tight linkage with the *Glu-B3* locus on the short arm of chromosome 1B and its involvement in gluten quality formation [41]. Based on the present data combined with published reports, a total of 21 alleles have been described at this locus. The *Gli-B1c* and *Gli-B1b* alleles, encoding γ-45 gliadins and considered desirable, were most characteristic of the European group (Supplementary Material Table S5). In contrast, the *Gli-B1a* allele, encoding γ-42 gliadins and associated with reduced gluten quality [7], remained common across all groups, with the highest frequency in the Eurasian group and the lowest in the European group. The *Gli-B1la* allele was detected in all regions, exhibiting pronounced geographic variation in frequency.

The *Gli-A2* locus exhibited exceptionally high allelic diversity. By combining literature data with the results of the present study, 26 alleles were identified, more than 80% of which were classified as rare (< 5%). The *Gli-A2a* and *Gli-A2o* alleles, typical of Ethiopian wheat [42], were present in all geographic groups, whereas a substantial proportion of alleles showed region-specific distributions (Supplementary Material Table S5). The highest number of rare alleles was recorded in the Eurasian group, indicating its role as a reservoir of genetic diversity.

For the *Gli-B2* locus, 44 previously described alleles and 7 novel alleles were identified. The *Gli-B2h* and *Gli-B2hk* alleles, which form highly similar gliadin blocks, occurred in most groups and likely share a close genetic origin and similar effects on agronomically important traits. Notably, half of the alleles detected in the Eurasian group were unique to this region. Despite the small sample size, the Australian group contained endemic alleles, highlighting pronounced regional specificity. Calculations of within-population diversity (*µ*), genetic diversity (*H*), and the proportion of rare alleles (*h*) showed that the highest *µ* values for the *Gli-1* and *Gli-2* loci were characteristic of the Eurasian group (Supplementary Material Table S6, Table 3).

As shown in Table 3, the Eurasian group exhibited the highest average intra-population diversity (*µ ± S_µ_*) for the *Gli-1* and *Gli-2* loci (12.12 ± 0.75). However, when examined per locus, the maximum µ value varied between regions. For example, for the *Gli-A1* locus, *µ* was higher in the Asian group (9.31 ± 0.91; *n* = 19) than in the Eurasian group (6.08 ± 0.44; *n* = 155), despite both groups having the same number of identified alleles (*n* = 11). This difference reflects the allele-frequency distribution: the Eurasian group was dominated by *Gli-A1g* (61.6%) and *Gli-A1eb* (17.7%), with the remaining nine alleles each below 5%, whereas the Asian group showed a more uniform distribution, with *Gli-A1b* and *Gli-A1c* at 26.3% and *Gli-A1mc* at 10.5%. Consequently, intra-population diversity is higher when allele frequencies are more evenly distributed. The additional diversity index, *h*, which equals 0 when alleles are equally frequent, further confirmed this pattern. For instance, the *Gli-A1* locus in the Eurasian group had *h* = 0.44 ± 0.04, indicating a notable imbalance in allele frequencies (Supplementary Material Table S6). Notably, smaller groups, such as the Asian accessions, often displayed higher *µ* values for *Gli-1* and *Gli-2* loci, emphasizing the influence of both sample size and allele distribution. To assess inter-population differentiation, the identity criterion (*I*) was calculated using the *r ± S_r_* indicator of genetic similarity (Table 4; Supplementary Material Table S7). When *I* exceeded the tabulated *χ^2^* value at a given significance level, differences between groups were considered significant [43].

While *I* values did not always surpass *χ*^2^, reflecting high-frequency shared alleles, the index effectively captured overall inter-population differences, although it is less sensitive to rare alleles. Using a stringent significance threshold of 1% (*p* < 0.01), differences were observed for several region pairs, with additional distinctions at 5% (*p* < 0.05) for selected loci. These results highlight that both allele frequency distributions and chosen significance levels critically influence the detection of genetic differentiation across durum wheat populations.

### 4.2. Glu-1 Loci

The *Glu-1* locus comprises two tightly linked genes encoding x- and y-type HMW glutenin subunits, which differ in molecular weight, isoelectric point, and cysteine residue content and collectively contribute to gluten polymer formation [4]. In durum wheat, the contribution of HMW-GS to grain and gluten quality is generally considered less pronounced than that of LMW-GS; nevertheless, numerous studies have reported significant but often inconsistent associations between specific *Glu-1* alleles and technological traits [44,45].

In the present study, no statistically significant relationships between individual HMW-GS and grain quality parameters were detected, including in breeding lines carrying subunits frequently reported as favourable (e.g., 1Ax1, 1Bx7 + 1By8) (Table 2). During electrophoretic identification, several HMW-GS with mobility differing from reference catalogue subunits were detected [27,33]. Comparable observations have been reported in Portuguese, Mediterranean, and other tetraploid wheat collections, where previously unclassified alleles of the *Glu-1* loci were described [46,47,48].

In particular, the *Glu-A1o* allele encoding subunit V has been associated with improved gluten quality relative to the common subunits 1Ax1 and 1Ax2 [4]. In the present work, subunits designated as 2** and 2*** based on electrophoretic mobility showed similarities to rare catalogue components; however, their correspondence to known alleles (including *Glu-A1o*) should be regarded as tentative. Definitive assignment requires molecular or proteomic characterization, which was beyond the scope of this study.

Allelic composition analysis showed that *Glu-A1b* and the null allele *Glu-A1c* predominated across all six geographic groups, a pattern consistent with previous reports and generally associated with reduced gluten quality [49,50]. Nevertheless, earlier studies demonstrated that the negative effect of *Glu-A1c* may be partially compensated by specific combinations of glutenin alleles at *Glu-B1*, *Glu-A3*, *Glu-B3*, and *Glu-B2* loci [47]. Such compensatory combinations were identified in a limited number of accessions in the present collection (Supplementary Material Table S1), emphasizing the importance of multi-locus interactions rather than single-locus effects.

At the *Glu-B1* locus, x-type subunits predominated across all regional groups, in agreement with their generally greater contribution to gluten strength compared with y-type subunits [51]. The most frequent subunit pairs (1Bx7 + 1By8, 1Bx6 + 1By8, and 1Bx20 + 1By20) correspond to those widely reported in durum wheat collections worldwide (Supplementary Material Table S8). While *Glu-B1b* (1Bx7 + 1By8) and *Glu-B1d* (1Bx6 + 1By8) are often associated with improved gluten properties, *Glu-B1e* (1Bx20 + 1By20) has been linked to reduced gluten strength and lower SDS sedimentation values [4,52]. Structural differences between these x-type subunits, including a cysteine-to-tyrosine substitution in the N-terminal domain of 1Bx20, have been proposed to impair gluten network formation by limiting disulfide bonding [3,53].

Population-level analysis revealed the highest intra-population diversity (*µ*) for HMW-GS in the European group (Table 3), reflecting both a large number of detected subunits and a more even allele frequency distribution. Elevated diversity at the *Glu-A1* and *Glu-B1* loci in European accessions is consistent with the long breeding history and extensive germplasm exchange within this region. In contrast, other regional groups were characterised by a smaller number of high-frequency alleles accompanied by a higher proportion of rare variants, resulting in lower effective diversity values. Elevated diversity at the *Glu-A1* and *Glu-B1* loci in European accessions is consistent with the long breeding history and extensive germplasm exchange within this region. In contrast, other regional groups were characterised by a smaller number of high-frequency alleles accompanied by a higher proportion of rare variants, resulting in lower effective diversity values (Table 5, Supplementary Material Table S7).

Pairwise identity analysis demonstrated statistically significant differences between most regional groups at the *Glu-1* loci. These differences were largely driven by variation in allele frequencies rather than by the presence of region-specific alleles, as the majority of accessions shared a limited set of common HMW-GS. When interpreted with this limitation in mind, the identity criterion provides evidence for moderate but significant population differentiation shaped primarily by unequal representation of widespread alleles.

Overall, the results indicate that although substantial polymorphism exists at the *Glu-1* loci in durum wheat, the direct effect of individual HMW-GS on grain quality traits is highly variable and strongly dependent on genetic context. The findings emphasise the need for integrative analyses combining HMW- and LMW-GS composition with molecular characterisation and standardised quality assessments to clarify the functional significance of *Glu-1* alleles in breeding programmes.

### 4.3. Glu-3 Loci

The LMW glutenin subunits of durum wheat are controlled by the *Glu-A3*, *Glu-B3*, and *Glu-B2* loci, which are closely linked to the gliadin loci *Gli-A1*, *Gli-B1*, and *Gli-B3*, respectively [4]. Although early catalogues reported a limited number of alleles at these loci, subsequent studies have demonstrated a substantial expansion of allelic diversity, particularly at *Glu-A3* and *Glu-B3* [7,18,47,54,55]. This increasing complexity highlights the difficulty of interpreting individual *Glu-3* effects without considering allelic combinations.

Previous studies have ranked *Glu-A3* alleles according to their contribution to gluten viscoelastic properties, with alleles *h*, *c*, *d*, and *a* generally associated with higher sedimentation values [56]. In agreement with earlier reports [57], our work confirmed a positive association between *Glu-A3a* (subunit 6) and sedimentation level. In contrast, the effects observed for *Glu-A3b* (subunits 8 + 9 + 13 + 16) on sedimentation level and gluten index differed from published data [56,57], suggesting that genotype × environment interactions may obscure direct allelic effects. Combinations of HMW-GS and LMW-GS have therefore been proposed as more reliable predictors of semolina quality than individual alleles [47]. In this context, the presence of *Glu-A3*, *Glu-B3*, and *Glu-B2* alleles *a* or *b*, together with *Glu-A1c* and *Glu-B1d*, has been associated with superior quality, whereas genotypes carrying *Glu-A1c* and *Glu-B1e+f* in combination with *Glu-A3a*, *Glu-B3a*, and *Glu-B2a+b* generally show reduced quality parameters [10,47]. This classification should be regarded as approximate, as grain quality is strongly influenced by environmental and agronomic factors [58,59].

Nevertheless, group-specific allelic variants were identified at the *Glu-3* loci. At *Glu-A3*, several alleles were unique to the European group, whereas alleles *a*, *b*, *c*, *d*, and *h* were detected across all geographic groups, with *Glu-A3a* being the most frequent overall (Supplementary Material Table S9).

The *Glu-B3* locus plays a central role in gluten quality through its control of LMW glutenin subunits, particularly those forming the LMW-2 pattern, which has been repeatedly associated with improved gluten strength [4,57,60]. In the present study, the negative effects of *Glu-B3b* [10] or *Glu-B3s* [55] on gluten index and SDSS volume were statistically confirmed (Table 2), consistent with earlier reports assigning a key positive role to *Glu-B3a* or *Glu-B3r* [10,55].

Using recommended combinations of HMW- and LMW-GS associated with high gluten quality [47,60,61], breeding lines were divided into two groups (Supplementary Material Table S2). Lines lacking *Glu-B3b* and carrying favourable *Glu-B3* alleles exhibited significantly higher mean values of gluten index and SDSS, while no differences were detected for protein or gluten content. These results support the practical value of combined HMW–LMW glutenin profiles for discriminating breeding material with contrasting gluten quality. Analysis of allelic distribution revealed that *Glu-B3* alleles *a*, *b*, *c*, and *d* were present in all geographic groups, although with different frequencies (Supplementary Material Table S9). The proportion of group-specific alleles was highest in European wheats, which also showed the greatest within-population variation (*μ*) and genetic diversity (*H*) at the *Glu-A3* and *Glu-B3* loci (Table 3).

Identity criterion (*I*) values for the *Glu-3* loci are presented in Table 5. While some group comparisons did not reach significance at the most stringent threshold, consistent trends toward differentiation became apparent when data were considered across loci, indicating moderate but non-random divergence among geographic groups.

**Figure 8:**
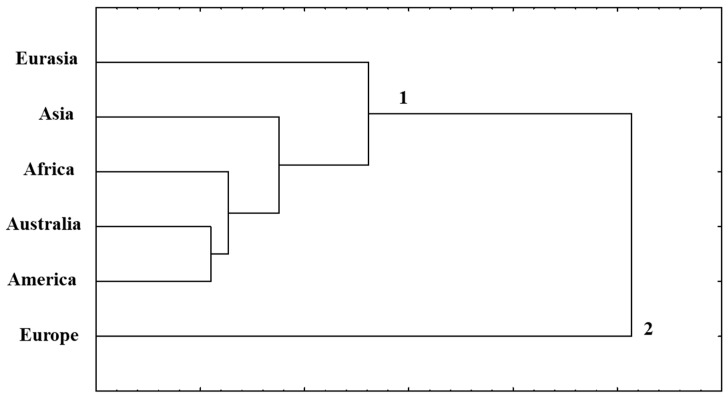
Cluster analysis based on the frequency (%) of alleles at the *Gli-1*, *Gli-2*, *Glu-1*, and *Glu-3* loci in durum wheat accessions according to their origin.

Finally, clustering based on the allelic composition of the *Gli-1*, *Gli-2*, *Glu-1*, and *Glu-3* loci (Figure 8) revealed a clear pattern of regional genetic differentiation. Overall, allelic overlap among geographical groups was low. The highest proportion of shared alleles was observed between Australian and American accessions, with 24 of the 264 identified alleles being common to both groups (9.0%). Comparisons involving African accessions showed a similar but slightly lower level of overlap (8.3%), while Eurasian and Asian accessions shared the smallest proportion of alleles with other regions (7.5%). The distinct clustering of Eurasian wheat is largely attributable to the accumulation of region-specific alleles at the *Gli-B2* locus, which were absent from other geographical groups and contributed substantially to its separation on the dendrogram. European wheat exhibited the highest degree of genetic distinctness, with 37.8% of alleles being unique to this region, reflecting pronounced variability at the *Glu-B1* and *Glu-B3* loci. Taken together, these results emphasize the central role of polymorphism at B-genome prolamin loci—particularly *Gli-B2*, *Glu-B1*, and *Glu-B3*—in shaping the regional structure of genetic diversity in durum wheat.

## 5. Conclusions

The identification of gliadin, HMW, and LMW glutenin alleles in durum wheat revealed substantial genetic diversity. Native and SDS-electrophoresis of prolamins remains an accessible and informative approach for analysing gluten protein composition. A set of 181 tetraploid wheat accessions, including 86 from Kazakhstan and 95 from the International germplasm collection, was studied for *Gli-1*, *Gli-2*, *Glu-1*, and *Glu-3* loci.

In total, 145 allele combinations of gliadin and glutenin were identified. Biochemical analysis and identification of HMW and LMW glutenin subunits were performed in 26 breeding lines. A positive association was observed between the gliadin component γ45 and the *Glu-A3a* allele, reflected in higher SDSS and GI values. In contrast, the *Glu-B3b* allele was associated with lower gluten quality. Statistical analyses revealed systematic differences in prolamin composition among genotypes from diverse ecological zones and geographic origins. These findings provide a basis for selecting durum wheat genotypes with potentially superior gluten quality in breeding programs.

## Figures and Tables

**Table 1:** Values of rare allele frequency (*h ± Sh*), genetic diversity (*H*), and within-population diversity (*μ ± Sμ*) at the *Gli-1*, *Gli-5*, *Glu-1*, and *Glu-3* loci.

Loci	Tetraploid Wheat Collection	*µ ± S*	*h ± S*	*H*
*Glu-A1*	Kazakhstan	3.66 ± 0.10	0.08 ± 0.02	0.69
	International	3.44 ± 0.05	0.31 ± 0.05	0.50
*Glu-B1*	Kazakhstan	5.04 ± 0.48	0.44 ± 0.05	0.45
	International	9.63 ± 0.59	0.26 ± 0.04	0.83
*Gli-A1*	Kazakhstan	6.43 ± 0.58	0.41 ± 0.05	0.64
	International	10.31 ± 0.72	0.31 ± 0.05	0.80
*Gli-B1*	Kazakhstan	5.46 ± 0.53	0.45 ± 0.05	0.51
	International	14.40 ± 0.84	0.24 ± 0.04	0.86
*Gli-A2*	Kazakhstan	6.60 ± 0.70	0.49 ± 0.05	0.48
	International	14.92 ± 1.06	0.32 ± 0.05	0.80
*Gli-B2*	Kazakhstan	18.91 ± 1.15	0.24 ± 0.04	0.88
	International	27.29 ± 1.54	0.22 ± 0.04	0.93
*Gli-B5*	Kazakhstan	1.83 ± 0.06	0.08 ± 0.03	0.34
	International	1.98 ± 0.01	0.01 ± 0.01	0.50
*Glu-A3*	Kazakhstan	5.04 ± 0.41	0.37 ± 0.05	0.65
	International	4.93 ± 0.33	0.30 ± 0.04	0.63
*Glu-B3*	Kazakhstan	5.79 ± 0.53	0.42 ± 0.05	0.63
	International	6.20 ± 0.34	0.23 ± 0.04	0.74
*Glu-B2*	Kazakhstan	1.80 ± 0.07	0.12 ± 0.03	0.32
	International	2.00 ± 0.02	0.01 ± 0.01	0.48

**Table 2:** Association analysis of high- and low-molecular-weight glutenin subunits and γ45 gliadin components with grain quality traits in 26 durum wheat breeding lines (2022–2024). *r_Q_*, Yule’s Q coefficient; *r_A_* (*χ^2^_f_*), Chi-square test value with Yates’ correction.

HMWG/Allele/γ-Gliadin	Stat. Index	Technological and Biochemical Traits			
		PC	GC	GI	SDSS
1Ax1	*r_A_ (χ^2^_f_)*	0.19 (2.81)	0.18 (2.71)	−0.01 (0.01)	0.02 (0.05)
	*r_Q_ ± S*	0.43 ± 0.19	0.41 ± 0.19	−0.03 ± 0.05	0.13 ± 0.27
1Bx7 + 1By8	*r_A_ (χ^2^_f_)*	0.09 (0.66)	0.15 (1.80)	−0.03 (0.07)	0.00 (0.00)
	*r_Q_ ± S*	0.37 ± 0.31	0.48 ± 0.15	−0.01 ± 0.31	−0.15 ± 0.41
*Glu-A3a*	*r_A_ (χ^2^_f_)*	0.17 (2.14)	0.00 (0.00)	0.49 (19.11)	0.42 (13.45)
	*r_Q_ ± S*	−0.39 ± 0.21	0.06 ± 0.23	0.85 ± 0.08	0.87 ± 0.08
*Glu-B3b*	*r_A_ (χ^2^_f_)*	0.11 (0.96)	0.09 (0.58)	0.26 (5.39)	0.37 (10.67)
	*r_Q_ ± S*	0.27 ± 0.21	−0.22 ± 0.22	0.55 ± 0.17	−0.84 ± 0.12
*Glu-B2b*	*r_A_ (χ^2^_f_)*	0.18 (2.61)	0.00 (0.00)	0.06 (0.33)	0.01 (0.01)
	*r_Q_ ± S*	0.48 ± 0.22	0.07 ± 0.25	0.22 ± 0.25	0.13 ± 0.30
*γ45*	*r_A_ (χ^2^_f_)*	0.08 (0.49)	0.00 (0.00)	0.52 (21.26)	0.59 (27.15)
	*r_Q_ ± S*	−0.24 ± 0.24	0.07 **±** 0.25	0.89 ± 0.07	0.93 ± 0.05

Note: If *χ^2^_f_* > *χ^2^_St_* = 3.84, and α = 5%, df = 1, then the association is statistically confirmed. If |rQ| > 0.3 and $\frac{\left|r_{Q}\right|}{S}=t>t_{St}=1.99$ and α = 5%, df = *n* − 2, then the association is statistically confirmed.

**Table 3:** Average rare allele frequency (*h ± S_h_*), genetic diversity (*H*), and within-population diversity (*μ ± S_μ_*) for the *Gli-1*, *Gli-2*, *Glu-1* and *Glu-3* loci.

Loci	Eurasia	Asia	Africa	Australia	Europe	America
*µ* ± *S_µ_*						
*Gli-1*, *Gli-2*	12.12 ± 0.75	10.68 ± 0.77	7.39 ± 0.65	3.93 ± 0.15	8.92 ± 0.56	4.46 ± 0.58
*Glu-1*	4.78 ± 0.31	4.93 ± 0.31	4.51 ± 0.24	5.22 ± 0.39	8.86 ± 0.47	3.53 ± 0.28
*Glu-3*	5.54 ± 0.42	4.59 ± 0.47	4.89 ± 0.43	5.30 ± 0.43	28.22 ± 1.41	4.21 ± 0.34
*h ± S_h_*						
*Gli-1*, *Gli-2*	0.39 ± 0.04	0.09 ± 0.07	0.12 ± 0.07	0.02 ± 0.07	0.32 ± 0.04	0.20 ± 0.11
*Glu-1*	0.29 ± 0.04	0.32 ± 0.05	0.33 ± 0.04	0.24 ± 0.06	0.39 ± 0.03	0.45 ± 0.04
*Glu-3*	0.38 ± 0.05	0.17 ± 0.09	0.36 ± 0.06	0.18 ± 0.07	0.33 ± 0.03	0.45 ± 0.04
*H*						
*Gli-1*, *Gli-2*	0.71	0.88	0.77	0.67	0.78	0.59
*Glu-1*	0.63	0.61	0.52	0.61	0.63	0.35
*Glu-3*	0.66	0.68	0.57	0.69	0.84	0.45

**Table 4:** Mean values of genetic similarity—*r ± S_r_* and identity criterion—*I (χ^2^_St_)* among durum wheat accessions based on the *Gli-1* and *Gli-2* loci across regions of origin.

Regions	Eurasia	Asia	Africa	Australia	Europe	America
Eurasia		0.51 ± 0.07	0.46 ± 0.08	0.54 ± 0.10	0.61 ± 0.04	0.64 ± 0.07
	0	176.5 (124.12)	144.7 (119.41)	57.25 (122.33)	635.2 (125.28)	90.92 (114.69)
Asia			0.56 ± 0.09	0.37 ± 0.10	0.51 ± 0.06	0.51 ± 0.09
		0	61.86 (93.21)	51.53 (83.51)	149.86 (105.2)	74.43 (83.51)
Africa				0.49 ± 0.12	0.52 ± 0.07	0.61 ± 0.11
			0	37.47 (63.69)	110.07 (93.22)	58.89 (62.43)
Australia					0.51 ± 0.07	0.41 ± 0.14
				0	58.01 (81.07)	45.82 (50.89)
Europe						0.51 ± 0.08
					0	125.65 (83.51)
America						0

Note: Chi-square *χ^2^* value was evaluated and is shown in brackets at the 1% significance level. If *I* > *χ^2^*, the differences are statistically significant.

**Table 5:** Mean values of the identity criterion (*I*) for durum wheat accessions based on the *Glu-1* (upper value) and *Glu-3* (lower value) loci across regions of origin.

Regions	Eurasia	Asia	Africa	Australia	Europe	America
Eurasia	0	61.36 (45.64)	207.10 (32.00)	59.65 (33.41)	229.28 (56.06)	235.14 (45.64)
		30.00 (33.41)	101.56 (40.29)	57.77 (34.81)	229.79 (127.63)	139.11 (41.64)
Asia		0	75.71 (30.58)	36.31 (33.41)	83.14 (56.06)	135.72 (36.19)
			16.51 (32.00)	22.48 (27.69)	43.92 (122.94)	32.70 (32.00)
Africa			0	53.69 (33.41)	79.54 (56.06)	93.92 (45.64)
				26.79 (33.41)	120.63 (128.80)	36.31 (38.93)
Australia				0	52.94 (57.34)	59.67 (37.57)
					61.18 (122.94)	29.72 (33.41)
Europe					0	170.06 (59.89)
						180.04 (129.97)
America						0

Note: Chi-square *χ^2^* value shown in brackets at the 1% significance level. If *I* > *χ^2^*, the differences are statistically significant.

## Data Availability

The original contributions presented in this study are included in the article/Supplementary Material. Further inquiries can be directed to the corresponding authors.

## Supplementary Materials

The following supporting information can be downloaded at: https://www.mdpi.com/article/10.3390/life16010157/s1. Table S1: Tetraploid wheat germplasm accessions used in the study; Table S2: Composition of high- and low-molecular-weight glutenin subunits and biochemical evaluation of breeding lines of Kazakhstan durum wheat (harvests of 2022–2025); Table S3: Frequencies (%) of Alleles at *Gli-1* and *Gli-2* loci in the International tetraploid wheat germplasm collection; Table S4: Tetraploid wheat grouped by eco-geographic origin; Table S5: Allele frequency (%) of *Gli-1* and *Gli-2* loci in International durum wheat accessions; Table S6: Values of rare allele frequency (*h ± S_h_*), genetic diversity (*H*), and within-population diversity (*μ ± S_μ_*) at the *Gli-1*, *Gli-5*, *Glu-1*, and *Glu-3* loci; Table S7: Similarity coefficient and Identity criterion; Table S8: Allele frequency (%) of *Glu-1* loci in International durum wheat accessions; Table S9: Allele frequency (%) of *Glu-3* loci in International durum wheat accessions.
